# Patients' Payments and Contributions

**Published:** 1916-11-18

**Authors:** 


### THE MODIFIED PAY SYSTEM.

### Patients' Payments and Contributions.

### I.—GENERAL CONSIDERATIONS.

A very large number of the hospitals in this country are at the present time receiving wounded and sick sailors and soldiers in respect of each of whom a grant of several shillings a day is made by the Government. The aggregate sum received for services rendered must be considerable, and most useful to help meet increased expenses. When the war is over it is not to be expected that the hospital budget will automatically revert to its former limits; indeed, it is unlikely that the cost of food, drugs, and other things largely used in hospitals will recede to anything like pre-war conditions for many years. What is almost certain is that while expenditure remains high, an important, though temporary, source of income will be taken away, and ways and means will have to be examined and a solution sought either in the direction of reducing expenditure or of increasing income. From the revenue point of view it may very well be that many hospitals will have to consider in some degree the adoption of the contributing principle, and especially will that principle appeal where its advantages have been so recently experienced.

#### Difficulties of an Altered System.

The voluntary system in this country, which has in the past been so firmly attached to the practice of giving freely everything in the shape of treatment and maintenance to the comparatively and actually poor, has earned such a reputation for excellence that doubtless it has been deemed hazardous to alter its character by any such change as the widespread introduction of contributing beds or wards. Moreover, the system has been opposed by practical difficulties, not the least of which is the position of the honorary medical staff, who, giving services gratuitously (as far as monetary reward is concerned), are naturally anxious that their exact position should be made clear to the patients under their charge, and that neither through ignorance nor misunderstanding should it be thought that their services are in any way remunerated through fees paid to the hospital. Another difficulty is that hospitals have not, as a rule, been built with a view to accommodate paying patients, and the idea of mixing free and contributing cases in the same wards is not always acceptable to hospital boards.

The justice of payment towards the cost of maintenance by the poorer patients who are, or whose families are, temporarily saved the cost of food, etc., seems unassailable. Within the last few months a hospital for children has examined the question, and in support of a proposal to require payment by suitable patients, two cases were reported by the lady almoner in illustration. It must be remembered that these were instances chosen to support a theory and must be accepted as such. One was that of an illegitimate child for whose support the parents had been paying ten shillings a week in a private home. The other was a case from a Poor-Law infirmary where the Guardians had enforced a payment for maintenance. In each instance it could be argued that free admission to a hospital would amount to a clear monetary gain by those responsible for the child's maintenance.

#### Provident Principles in Voluntary Hospitals.

The veteran secretary of the Central London Throat and Ear Hospital, in dealing with this subject in a pamphlet entitled "Provident Principles in Voluntary Hospitals," says:

"There is a wide difference between converting a charitable hospital into a commercial undertaking, as some fear this contributing system will lead to, and asking a patient to give a small contribution towards the expense in outlay in drugs and food; for the skill in medical treatment and hospital nursing will always remain as a great boon to which the benevolent may be proud to contribute, and which no small money payment from the patients, such as I advocate, can either liquidate or diminish."

But it is not always that it is a question of considering the needs of the very poor. Great convenience would accrue to the middle classes (possibly to the upper classes, too) by some alternative to the private nursing home.

These are changing times; what seemed revolutionary yesterday is accepted as natural and reasonable to-day. The moment is clearly opportune to review what has been done in the past in the matter of obtaining contributions from or on behalf of patients, what are present arrangements in this respect, and which are the best methods of commencing the system in hospitals where hitherto no effort has been made to collect revenue in this form or to provide for sick persons whose financial position is not suited to gratuitous relief in a charitable institution.

#### Systems of Other Countries.

In other countries, speaking generally, the contributing system has been in force for many years, and in a form which is neither desirable nor necessary as applied to conditions in the British Islands. Abroad it may be said that charitable feeling finds expression in the building and equipment of the great curative institutions, and their upkeep is left largely to the State or to the municipalities who support, or help to support, needy persons whilst in the wards, whilst provision is made for those who can afford to defray the cost of maintenance and treatment at rates arranged to meet their varying financial status. Such is the main underlying principle, whilst, of course, in detail the arrangements and rules of the hospitals vary in different lands. As a European example, Sweden and Norway may be quoted; here the annual budget of a large institution is submitted to Parliament, who vote any deficiency. The Poor-Law authorities pay for the lowest class in wards of four to ten beds, where the patients receive everything necessary to their cure, including food of a plain character; in the second class the patients themselves contribute in wards of two to three beds, with more liberal diet; and in the third or highest grade the patients are given single rooms and entirely defray the cost of treatment and maintenance.

In some of our Colonies the system is very similar. Public benevolence provides the building, and the patients, or those paying for them, make secure a large part of the cost of maintenance. Contributions are made towards current expenses in the shape of donations and subscriptions, but where it is clearly a case of private generosity relieving the rates and taxes the volume of such support has a tendency to decline.

Our own voluntary system is so dear to the hearts of the people, and an outlet for charity and practical sympathy is so much in keeping with the British character, that a change to Continental or Colonial methods would be neither popular nor desirable. But there is a wide difference between the system of other countries and one which would bring home to the poor patient and the friends of the poor patient a sense of their responsibilities, and at the same time make moderate provision for the better-to-do classes. Such a principle has governed the management of our cottage hospitals for many years, and the arrangements have been uniformly acceptable to the patients of all classes and to the supporters of the hospital. As regards larger hospitals, what has been done in this direction has been neither extensive nor similar in the nature of the arrangements. In what main features the methods vary will be indicated in another article, but before arriving at that aspect of the subject we will deal briefly with the history of contributing beds in this country.

### Some Pioneer Contributing Schemes.

Many years ago it was realised by those interested in hospitals that for want of suitable arrangements and accommodation many persons who would benefit by and should receive hospital treatment were excluded because of financial status, or, if admitted to the existing hospitals, created a form of abuse which could only in very pressing cases of emergency be justified to the supporters of the institutions. In 1840 an influential body of men, to meet the difficulty, founded a Sanatorium or Home in Sickness at Devonshire Place House, New Road. The institution started under the best auspices; the Prince Consort accepted the office of President, and the chief organiser was the late Dr. Southwood Smith. The building and equipment were provided by public subscription, and it was hoped that the contributions of patients would supply sufficient funds for upkeep. Although the weekly cost of each patient worked out at less than £2 2s. per week the institution was short-lived, and at the end of two years closed its doors, having made a loss of £350 a year. It was claimed that the scheme was killed by success, and that there were too many applicants for the small number of available beds. The history of the concern is rather obscure, but it seems fairly probable that an institution with every chance of success perished for want of efficient business management.

A more successful enterprise was inaugurated through the Home Hospital movement in 1877, a healthy outward sign of which is still with us in the shape of Fitzroy House, where, in an establishment of thirty beds, some four to five hundred patients, attended by their own medical men, find at a cost of from £4 4s. a week a substitute, in the shape of a private hospital, for the ordinary nursing home, and do so in confidence that a high degree of efficiency is not a mere matter of chance. That this Home Hospital movement was not extended is largely the fault of the voluntary hospitals, for the movement was intended to lead the way to the solving of the whole question of providing accommodation within the means of middle-class patients when urgently in need of hospital treatment. The only whole-hearted effort to work in this badly neglected field of enterprise and benevolence has been on the part of St. Thomas's Hospital, where the excellent arrangements of the separate Nursing Home for Paying Patients have been appreciated by thousands of persons of moderate means who are, in sickness, quite as worthy of consideration as the very poor. With the details of this Home we shall deal at a later date.

### Extent of Present Accommodation.

These illustrations are of institutions outside, or attached to, hospitals. There are, of course, beds for contributing patients in hospitals, and arrangements under which patients in general wards contribute varying sums according to means. From the Statistical Report for 1914 of King Edward's Hospital Fund for London it is discovered that the total number of the beds dealt with in the report is 10,939, and that 622, or 5.7 per cent., are classed as "paying beds." Of the income of hospitals as shown in "Burdett's Hospitals and Charities" it is learned that revenue from patients' payments in two chosen years was as follows:—

| London Hospitals:— | | 1892 | | 1913 |
|---|---|---|---|---|
| With Medical Schools ... | (13) | £4,921 | (12) | £10,736 |
| Without Medical Schools | (11) | £1.450 | (14) | £7,682 |

From these figures it will be gathered that the justice and convenience of payments by patients have been accepted in at least some quarters, but in examining any statistics of this kind it must be remembered that in recent years the class of the community making use of hospitals has changed, because the nature of the provision for the more destitute poor has been enormously improved by Poor-Law and municipal authorities. The hospitals now largely help a different class amongst the workers of the community, and to meet the altered and improved status of a majority of the patients the conditions under which relief is given must, in justice to the supporters of the voluntary system, be examined, and, if need be, altered to meet the exigencies of the times.

